# Supplementary material for: Environmentally co‐occurring mercury resistance plasmids are genetically and phenotypically diverse and confer variable context‐dependent fitness effects
Source: Environ Microbiol. 2015 Jun 25;17(12):5008–22. doi: 10.1111/1462-2920.12901 (PMC4989453; doi:10.1111/1462-2920.12901)
Supplement: Supplementary file 1 — Fig. S1. Relative fitness of different plasmid‐bearing transconjugants when grown in competition with plasmid free. Several GmR‐labelled and SmR‐labelled transconjugants were tested under low, intermediate and high levels of mercury and relative fitness in KB was plotted as in Fig. 4. One randomly selected transconjugant (coloured in red) was chosen for subsequent experiments. Fig. S2. (a) PCRs using DNA extracted from pQBR44‐bearing bacteria as a template were performed with different primer combinations and were separated on a 1% agarose gel. Stylized diagrams describing the predicted topologies of the templates given a product are shown to the left of each gel. mus‐9 is a positive control for template (Ramos‐Gonzalez et al., 2005). (b) The predicted structure of pQBR44 given the PCR results. Fig. S3. Regions of similarity between the pQBR plasmids and with previously sequenced genomes. As shown in Fig. 2, except the matches for each plasmid are shown separately for clarity. In a clockwise direction, the pQBR44 contigs are pQBR44.2, pQBR44.1; the P. stutzeri B1SMN1 contigs are 16 (reversed), 32 (reversed) and 9; and the P. maculicola ES4326 contigs are 6.12, 6.13, 6.14, 6.2, 6.3, 6.4, 6.5, 6.6, 6.8, 6.9, 6.10 and 6.11. Image was drawn using Circos (Krzywinski et al., 2009). Fig. S4. Loss of plasmids over time. Plasmid‐bearing clones were used to inoculate KB microcosms without mercury. Every 48 h a sample of culture was diluted 1:100 into fresh KB. Plasmid frequency was regularly assessed by replica plating colonies onto KB agar + 100 μM HgCl2. Fig. S5. Relative fitness of test plasmid bearers when grown in competition with reference plasmid bearers in KB broth. As the left‐hand panel of Fig. 5 except plots are separated by marker orientation, and individual replicates are shown. Fig. S6. Relative fitness of plasmid bearers when grown in competition with plasmid free in KB broth (left) and potting soil (right) microcosms. As Fig. 4 except fitness was calculated as [file EMI-17-5008-s001.zip › PlasmidGenomesImageChanges.docx]

Modifications to images:

Figure 1: It was noticed that histogram colouring was incorrectly aligned in the original submission. This has been corrected in the revision.

Figure 3: On the suggestion of Reviewer 1, all points have been converted to filled circles.

Figure S4: On the suggestion of Reviewer 3, the x-axis has been converted into ‘days’ rather than ‘transfers’.

Figure S6: Figure S6 has been added in response to Reviewer 3.
