# Supplementary material for: Environmentally co‐occurring mercury resistance plasmids are genetically and phenotypically diverse and confer variable context‐dependent fitness effects
Source: Environ Microbiol. 2015 Jun 25;17(12):5008–22. doi: 10.1111/1462-2920.12901 (PMC4989453; doi:10.1111/1462-2920.12901)
Supplement: Supplementary file 1 — Fig. S1. Relative fitness of different plasmid‐bearing transconjugants when grown in competition with plasmid free. Several GmR‐labelled and SmR‐labelled transconjugants were tested under low, intermediate and high levels of mercury and relative fitness in KB was plotted as in Fig. 4. One randomly selected transconjugant (coloured in red) was chosen for subsequent experiments. Fig. S2. (a) PCRs using DNA extracted from pQBR44‐bearing bacteria as a template were performed with different primer combinations and were separated on a 1% agarose gel. Stylized diagrams describing the predicted topologies of the templates given a product are shown to the left of each gel. mus‐9 is a positive control for template (Ramos‐Gonzalez et al., 2005). (b) The predicted structure of pQBR44 given the PCR results. Fig. S3. Regions of similarity between the pQBR plasmids and with previously sequenced genomes. As shown in Fig. 2, except the matches for each plasmid are shown separately for clarity. In a clockwise direction, the pQBR44 contigs are pQBR44.2, pQBR44.1; the P. stutzeri B1SMN1 contigs are 16 (reversed), 32 (reversed) and 9; and the P. maculicola ES4326 contigs are 6.12, 6.13, 6.14, 6.2, 6.3, 6.4, 6.5, 6.6, 6.8, 6.9, 6.10 and 6.11. Image was drawn using Circos (Krzywinski et al., 2009). Fig. S4. Loss of plasmids over time. Plasmid‐bearing clones were used to inoculate KB microcosms without mercury. Every 48 h a sample of culture was diluted 1:100 into fresh KB. Plasmid frequency was regularly assessed by replica plating colonies onto KB agar + 100 μM HgCl2. Fig. S5. Relative fitness of test plasmid bearers when grown in competition with reference plasmid bearers in KB broth. As the left‐hand panel of Fig. 5 except plots are separated by marker orientation, and individual replicates are shown. Fig. S6. Relative fitness of plasmid bearers when grown in competition with plasmid free in KB broth (left) and potting soil (right) microcosms. As Fig. 4 except fitness was calculated as [file EMI-17-5008-s001.zip › PlasmidGenomesSupportingTextRevised.docx]

**SUPPORTING INFORMATION**

**Figure Legends**

**Figure S1.** Relative fitness of different plasmid-bearing transconjugants when grown in competition with plasmid-free. Several Gm^R^-labelled and Sm^R^ labelled transconjugants were tested under low, intermediate and high levels of mercury and relative fitness in KB plotted as in Figure 4. One randomly selected transconjugant (coloured in red) was chosen for subsequent experiments.

**Figure S2.** (a) PCR reactions using DNA extracted from pQBR44-bearing bacteria as a template were performed with different primer combinations and were separated on a 1% agarose gel. Stylised diagrams describing the predicted topologies of the templates given a product are shown to the left of each gel. mus-9 is a positive control for template (Ramos-Gonzalez et al., 2005) (b) The predicted structure of pQBR44 given the PCR results.

**Figure S3.** Regions of similarity between the pQBR plasmids and with previously sequenced genomes. As Figure 2, except the matches for each plasmid are shown separately for clarity. In a clockwise direction, the pQBR44 contigs are pQBR44.2, pQBR44.1; the *P. stutzeri* B1SMN1 contigs are 16 (reversed), 32 (reversed) and 9; and the *P. maculicola* ES4326 contigs are 6.12, 6.13, 6.14, 6.2, 6.3, 6.4, 6.5, 6.6, 6.8, 6.9, 6.10, 6.11. Image was drawn using Circos (Krzywinski et al., 2009).

**Figure S4.** Loss of plasmids over time. Plasmid-bearing clones were used to inoculate KB microcosms without mercury. Every 48 hours a sample of culture was diluted 1:100 into fresh KB. Plasmid frequency was regularly assessed by replica plating colonies onto KB agar + 100 µM HgCl_2_.

**Figure S5.** Relative fitness of test plasmid-bearers when grown in competition with reference plasmid-bearers in KB broth. As the left-hand panel of Figure 5 except plots are separated by marker orientation, and individual replicates are shown.

**Figure S6.** Relative fitness of plasmid-bearers when grown in competition with plasmid-free in KB broth (left) and potting soil (right) microcosms. As Figure 4 except fitness was calculated as the selection rate constant s = (log(test_start_/test_end_) – log(reference_start_/reference_end_).

**Table S1.** To calculate whether our method of retrieving bacteria from soil was biased towards or against plasmid carriers, soil competitions (n = 4) with no added Hg(II) for each plasmid were set up and recovered 1 h after inoculation. Selection rate constants (s = (log(test_start_/test_end_) – log(reference_start_/reference_end_)) / time) were calculated as a measure of differential retention of bacterial genotypes by the soil, and one-sample t-tests performed. The selection rate was not significantly different from zero for any of the plasmids tested, suggesting that the relative fitness of the plasmid-bearers is more likely due to differential growth than biased retrieval.

**Table S2. Putative functions of predicted CDS.** ^a^As defined by RAST (Aziz et al., 2008). ^b^Top BLASTP hit. Only hits that were in the RefSeq database and had an E-value of > 0.01 were considered. ^c^Regions were determined by hand based on putative groups of functionally linked genes and comparisons with previously annotated regions.

**Table S3. Primer sequences used in this study.** Primers mus–9 (3+) and mus–9 (4–) were from Ramos-Gonzalez et al. (2005).

**Supporting Information: details of sequencing and annotation**

Plasmid pQBR57 and pQBR55 were sequenced in both *P. putida* UWC1 (McClure et al., 1989, ‘Pp reads’) and *P. fluorescens* SBW25-Gm^R^ (‘Pf reads’); the non-conjugative pQBR44 was sequenced only in *P. putida* UWC1. For each of pQBR55 and pQBR57, plasmid sequences were obtained by subtracting from the Pf reads those that matched the *P. fluorescens* SBW25 genome (ENA number AM181176 (Silby et al., 2009)) and assembling the remainder using SPAdes v2.5.1 with kmers 21, 33, 55 and 77 (Bankevich et al., 2012). The code used is available at <https://github.com/scottishwormboy/LIMS3104>. Sequences were verified by comparison with the corresponding Pp reads. For pQBR55, scaffolds were closed by identifying overlaps with previously obtained sequences (ENA number AJ421512). To close pQBR57, the product of a PCR reaction using primers pQBR57_306770F and pQBR57_176R (see Supporting Information Table S3) was Sanger sequenced and found to be consistent with the contig sequence.

For pQBR44, scaffolds were obtained by subtracting reads that matched the preliminary *P. putida* UWC1 genome (assembled from the Pp reads once the plasmid-matching reads were removed) and assembling the remainder using SPAdes. Unlike pQBR55 and pQBR57, the ends of the pQBR44 contigs did not overlap preventing this sequence from being closed. Further inspection revealed that each contig had a 77 bp inverted sequence at each end. BLASTN analysis of these sequences against the nr database showed that these were a match for a region of the *P. putida* KT2440 (of which the UWC1 strain used in this study is a derivative (McClure et al., 1989)) genome that is annotated as a ~40 kbp transposable element (ENA number AE015451, between 6128576 and 6152974, (Weinel et al., 2002)). We therefore considered the hypothesis that pQBR44 has acquired one or more copies of this transposon which have inserted between the assembled contigs. Primers were designed to amplify the putative junctions between contigs and transposon(s) (see Supporting Information Table S3), and PCR was run under the following conditions: denaturation at 95°C for 5 minutes; 30 cycles of 95°C for 30 seconds, 67°C for 30 seconds, 72°C for 1 minute; final extension at 72°C for 5 minutes. Supporting Figure S2a shows the products of the different reactions alongside a stylized diagram of the junction that was being tested. The products of reactions marked with asterisks were sequenced and yielded the expected sequence. Compiling this data suggests a plasmid with two copies of the transposon as shown in Supporting Figure S2b. This hypothesis is additional supported by coverage data, in that aligning the pQBR44 sample reads to the KT2440 genome yields coverage in the region 6128576–6152974 bp approximately 3x that of the surrounding region (not shown).

Notes and inferences obtained using InterProScan–5 (Zdobnov and Apweiler, 2001), BLASTP 2.2.28+ (Altschul et al., 1997) against the NCBI nr database (retrieved 19th October 2013), PSORTb–3.0.2 (Yu et al., 2010), PHOBIUS–1.01 (Kall et al., 2007) and SignalP–4.1 (Bendtsen et al., 2004) were added to each CDS using custom Python scripts (code at <https://gist.github.com/jpjh/9807c0744d182022726c>).

**SUPPORTING REFERENCES**

Altschul, S.F., Madden, T.L., Schäffer, A.A., Zhang, J., Zhang, Z., Miller, W., and Lipman, D.J. (1997) Gapped BLAST and PSI-BLAST: a new generation of protein database search programs. *Nucleic Acids Research* **25**: 3389–3402.

Aziz, R.K., Bartels, D., Best, A.A., DeJongh, M., Disz, T., Edwards, R.A., et al. (2008) The RAST Server: Rapid Annotations using Subsystems Technology. *BMC Genomics* **9**: 75.

Bankevich, A., Nurk, S., Antipov, D., Gurevich, A.A., Dvorkin, M., Kulikov, A.S., et al. (2012) SPAdes: A New Genome Assembly Algorithm and Its Applications to Single-Cell Sequencing. *Journal of Computational Biology* **19**: 455–477.

Bendtsen, J.D., Nielsen, H., Heijne, von, G., and Brunak, S. (2004) Improved prediction of signal peptides: SignalP 3.0. *Journal of Molecular Biology* **340**: 783–795.

Kall, L., Krogh, A., and Sonnhammer, E.L.L. (2007) Advantages of combined transmembrane topology and signal peptide prediction--the Phobius web server. *Nucleic Acids Research* **35**: W429–W432.

Krzywinski, M., Schein, J., Birol, I., Connors, J., Gascoyne, R., Horsman, D., et al. (2009) Circos: an information aesthetic for comparative genomics. *Genome Res.* **19**: 1639–1645.

McClure, N.C., Weightman, A.J., and Fry, J.C. (1989) Survival of Pseudomonas putida UWC1 containing cloned catabolic genes in a model activated-sludge unit. *Applied and Environmental Microbiology* **55**: 2627–2634.

Ramos-Gonzalez, M.I., Campos, M.J., Ramos, J.L., and Espinosa-Urgel, M. (2005) Characterization of the Pseudomonas putida Mobile Genetic Element ISPpu10: an Occupant of Repetitive Extragenic Palindromic Sequences. *Journal of Bacteriology* **188**: 37–44.

Silby, M.W., Cerdeño-Tárraga, A.M., Vernikos, G.S., Giddens, S.R., Jackson, R.W., Preston, G.M., et al. (2009) Genomic and genetic analyses of diversity and plant interactions of Pseudomonas fluorescens. *Genome Biol* **10**: R51.

Weinel, C., Nelson, K.E., and Tümmler, B. (2002) Global features of the Pseudomonas putida KT2440 genome sequence. *Environmental Microbiology* **4**: 809–818.

Yu, N.Y., Wagner, J.R., Laird, M.R., Melli, G., Rey, S., Lo, R., et al. (2010) PSORTb 3.0: improved protein subcellular localization prediction with refined localization subcategories and predictive capabilities for all prokaryotes. *Bioinformatics* **26**: 1608–1615.

Zdobnov, E.M. and Apweiler, R. (2001) InterProScan--an integration platform for the signature-recognition methods in InterPro. *Bioinformatics* **17**: 847–848.
