# Supplementary material for: Environmentally co‐occurring mercury resistance plasmids are genetically and phenotypically diverse and confer variable context‐dependent fitness effects
Source: Environ Microbiol. 2015 Jun 25;17(12):5008–22. doi: 10.1111/1462-2920.12901 (PMC4989453; doi:10.1111/1462-2920.12901)
Supplement: Supplementary file 1 — Fig. S1. Relative fitness of different plasmid‐bearing transconjugants when grown in competition with plasmid free. Several GmR‐labelled and SmR‐labelled transconjugants were tested under low, intermediate and high levels of mercury and relative fitness in KB was plotted as in Fig. 4. One randomly selected transconjugant (coloured in red) was chosen for subsequent experiments. Fig. S2. (a) PCRs using DNA extracted from pQBR44‐bearing bacteria as a template were performed with different primer combinations and were separated on a 1% agarose gel. Stylized diagrams describing the predicted topologies of the templates given a product are shown to the left of each gel. mus‐9 is a positive control for template (Ramos‐Gonzalez et al., 2005). (b) The predicted structure of pQBR44 given the PCR results. Fig. S3. Regions of similarity between the pQBR plasmids and with previously sequenced genomes. As shown in Fig. 2, except the matches for each plasmid are shown separately for clarity. In a clockwise direction, the pQBR44 contigs are pQBR44.2, pQBR44.1; the P. stutzeri B1SMN1 contigs are 16 (reversed), 32 (reversed) and 9; and the P. maculicola ES4326 contigs are 6.12, 6.13, 6.14, 6.2, 6.3, 6.4, 6.5, 6.6, 6.8, 6.9, 6.10 and 6.11. Image was drawn using Circos (Krzywinski et al., 2009). Fig. S4. Loss of plasmids over time. Plasmid‐bearing clones were used to inoculate KB microcosms without mercury. Every 48 h a sample of culture was diluted 1:100 into fresh KB. Plasmid frequency was regularly assessed by replica plating colonies onto KB agar + 100 μM HgCl2. Fig. S5. Relative fitness of test plasmid bearers when grown in competition with reference plasmid bearers in KB broth. As the left‐hand panel of Fig. 5 except plots are separated by marker orientation, and individual replicates are shown. Fig. S6. Relative fitness of plasmid bearers when grown in competition with plasmid free in KB broth (left) and potting soil (right) microcosms. As Fig. 4 except fitness was calculated as [file EMI-17-5008-s001.zip › PlasmidGenomesTextRevised_v5.1_Tracked.docx]

**Environmentally co-occurring mercury resistance plasmids are genetically and phenotypically diverse and confer variable context-dependent fitness effects**

James P. J. Hall^a1^, Ellie Harrison^a^, Andrew K. Lilley^b^, Steve Paterson^c^, Andrew J. Spiers^d^, Michael A. Brockhurst^a^

^a^Department of Biology, Wentworth Way, University of York, York, YO10 5DD, United Kingdom

^b^Pharmaceutical Science Research Division, King’s College London, Franklin-Wilkins Building, 150 Stamford Street, London, SE1 9NH, United Kingdom

^c^Institute of Integrative Biology, University of Liverpool, Liverpool, L69 7ZB, United Kingdom

^d^The SIMBIOS Centre, School of Science, Engineering and Technology, Abertay University, Dundee, DD1 1HG, United Kingdom

^1^To whom correspondence should be addressed. Tel: +44 1904 32 8683. Fax: +44 01904 32 8505. e-mail: [james.hall@york.ac.uk](mailto:james.hall@york.ac.uk)

Running title: Context-dependent effects of pQBR plasmid carriage

**Abstract**

Plasmids are important mobile elements that can facilitate genetic exchange and local adaptation within microbial communities. We compared the sequences of four co-occurring pQBR-family environmental mercury resistance plasmids and measured their effects on competitive fitness of a *Pseudomonas fluorescens* SBW25 host, which was isolated at the same field site. Fitness effects of carriage differed between plasmids and were strongly context dependent, varying with medium, plasmid status of competitor and levels of environmental mercury. The plasmids also varied widely in their rates of conjugation and segregational loss. We found that few of the plasmid-borne accessory genes could be ascribed functions, although we identified a putative chemotaxis operon, a type IV pilus-encoding cluster, and a region encoding putative arylsulfatase enzymes, which were conserved across geographically distant isolates. One plasmid, pQBR55, conferred the ability to catabolise sucrose. Transposons, including the mercury resistance Tn5042, appeared to have been acquired by the different pQBR plasmids by recombination, indicating an important role for horizontal gene transfer in the recent evolution of pQBR plasmids. Our findings demonstrate extensive genetic and phenotypic diversity amongst co-occurring members of a plasmid community and suggest a role for environmental heterogeneity in the maintenance of plasmid diversity.

**Introduction**

Horizontal gene transfer (HGT) is a central process in microbial evolution and ecology (Gogarten et al., 2005). Conjugative plasmids are widespread agents of HGT (Norman et al., 2009). These genetic elements are physically separate from the host chromosome, control their own replication, and encode DNA secretion systems enabling their transfer between bacterial hosts (Smillie et al., 2010). Rich plasmid communities have been isolated from a range of natural environments, including wastewater treatment plants, bovine rumen, fresh water, and soil (Sentchilo et al., 2013; Brown Kav et al., 2012; Brown et al., 2013, Lilley et al., 1996; Heuer and Smalla, 2012). Plasmids have been implicated in the dissemination of ecologically important traits, such as resistance to toxins and metabolism of novel substrates, which underpins environmental adaptation and facilitates colonisation of new habitats (Norman et al., 2009). Plasmids are hugely diverse in terms of their size (Smillie et al., 2010) and the genes that they contain; a large number of sequenced plasmid genes have no similarity to previously sequenced genes and a high proportion are of unknown function (e.g. Tett et al., 2007; Sentchilo et al., 2013; Xiong et al., 2013). Plasmids represent a vast pool of genetic diversity on which bacterial communities can draw, and understanding plasmid biology is central to understanding microbial evolution and ecology.

Plasmids are part of a continuum of mobile genetic elements (MGE) that gain and lose functions by recombination with the host chromosome and with one another, often resulting in a modular, mosaic genetic composition (Toussaint and Merlin, 2002). Plasmid-borne genes are often divided into ‘backbone’ genes and ‘accessory’ genes, with the former involved in vertical and horizontal transmission of the plasmid, and the latter encoding traits beneficial to the bacterial host (Norman et al., 2009). Since plasmids depend on host processes to survive and spread, the relationship between plasmid and host can be parasitic, with plasmids inflicting a net cost to their host through burdens such as plasmid DNA replication and expression of transfer-related genes (Baltrus, 2013). However, the benefits of expressing plasmid-borne traits might outweigh these costs, resulting in aligned fitness interests between plasmid and host.

The physical and chemical environment, known to affect symbioses in other ecological systems (Wolinska and King, 2009; Chamberlain et al., 2014), may determine the nature of the host-plasmid symbiosis and affect the fate of plasmids. Previous studies have shown that the costs and benefits of plasmid carriage to a host can be affected both by extrinsic factors such as availability of resources and exposure to antibiotics or toxic metals (Platt et al., 2012; Gullberg et al., 2014), and intrinsic factors such as gene expression and the specific combination of plasmid and bacterium (Dahlberg and Chao, 2003; De Gelder et al., 2007). However, there have been relatively few studies measuring host-plasmid fitness directly, or how this relationship varies across environmental gradients (Ellis et al., 2007; Gullberg et al., 2014).

A collection of 136 ‘pQBR’ mercury resistance (Hg^R^) plasmids, representing a naturally coexisting plasmid community, were previously isolated from the sugar beet phytosphere of an uncontaminated pristine pasture at Wytham in Oxfordshire, UK (Lilley et al., 1994; 1996; Lilley and Bailey, 1997b). Several of these plasmids have been the subject of extensive experiments characterising replication (Viegas et al., 1997; Turner et al., 2002), *in planta* colonisation and persistence (Lilley and Bailey, 1997a), and survival in spatially structured or genetically mixed communities (Slater et al., 2008; Ellis et al., 2007). One plasmid, pQBR103, was previously sequenced and found to contain a large number of genes with no known function, hinting at the diversity of uncharacterised genetic information present in the phytosphere and soil (Tett et al., 2007). Furthermore, Raman spectral analysis can differentiate between pQBR103-containing and plasmid-free bacterial cells grown on leaf and plant-conditioned soil washes, suggesting that cell physiology and metabolism are affected by plasmid carriage (Ude et al., 2007).

The pQBR plasmids represent an environmentally-relevant model system for understanding the ecology and evolution of natural conjugative plasmids. The aim of this study was to determine and compare the sequences of three pQBR plasmids, pQBR44, pQBR55, pQBR57, with pQBR103, and measure the effects of their carriage on the phenotype and fitness of a model pseudomonas host, *P. fluorescens* SBW25, under a range of conditions. These four plasmids were selected as representatives of the most common plasmid groups identified at Wytham using restriction fragment length polymorphism (RFLP) (Tett et al., 2007). *P. fluorescens* SBW25 was isolated independently from sugar beet at the same site (Rainey and Bailey, 1996). To directly test the effects of plasmid carriage we conjugated the plasmids into *P. fluorescens* SBW25 and compared their phenotypes. Our results show variation among plasmids in their effects on host fitness, that plasmid acquisition is likely to impose a net cost under most circumstances, but that the bacterial-plasmid symbiosis is strongly influenced by environmental context.

**Materials and Methods**

**Strains and plasmids**

*Pseudomonas fluorescens* SBW25 (Rainey and Bailey, 1996) was labelled with mini-Tn7 gentamicin resistance (Gm^R^) and streptomycin resistance *lacZ* (Sm^R^*lacZ*) cassettes according to the method of Lambertsen et al. (2004) to produce *P. fluorescens* SBW25-Gm^R^ and *P. fluorescens* SBW25-Sm^R^*lacZ*. An SBW25-*lacZ* strain (from Zhang and Rainey, 2007), which carries the *lacZ* marker, was also used. Plasmids originally isolated in *P. putida* UWC1 (pQBR55 and pQBR57; Lilley et al., 1996) and *P. fluorescens* SBW25 (pQBR103; Lilley and Bailey, 1997b) were transferred into the labelled *P. fluorescens* SBW25 lines by conjugation, and were selected using mercury and the appropriate antibiotic.

**Microcosms**

Bacteria were grown in modified King’s B medium (KB: 10 g glycerol, 1.5 g MgSO_4_.7H_2_O, 1.5 g K_2_HPO_4_.3H_2_O, 20 g Proteose peptone No. 3 (BD Company) l^–1^), supplemented with antibiotics (30 µg/ml gentamicin or 250 µg/ml streptomycin), 10–60 µM HgCl_2_ (Hg(II)) and 1.2% w/v agar, where required. Inocula for experiments were provided by 18 h overnight shaken cultures incubated at 28°C. Loosely lidded Universal bottles (30 ml) were used for microcosms: KB microcosms contained 6 ml KB, soil microcosms contained 10 g of twice-autoclaved John Innes #2 potting soil with ~25% w/v water content and were mixed with 800 µl 0–4 mM Hg(II) and allowed to equilibrate for 1 h before use.

**Competitive fitness assays**

Competitive fitness assays were initiated with 1:1 mixtures of independent overnight cultures of test and reference strains. Plasmid-bearing Gm^R^ test strains competed against plasmid-free SBW25-*lacZ*, whereas for plasmid-plasmid assays plasmid-bearing SBW25-Gm^R^ competed against plasmid-bearing SBW25-Sm^R^*lacZ*. Viable numbers of bacteria were estimated by spreading dilutions of culture on KB plates containing 50 µg/ml X-Gal. Relative fitness was calculated from these data as the ratio of Malthusian parameters W = ln(test_start_/test_end_) / ln(reference_start_/reference_end_) (Lenski et al., 1991). Assays in KB microcosms were initiated with 60 µl samples of the mixtures (approximately 1 x 10^8^ cfu) and incubated for 48 h shaking at 170 rpm at 28°C before assay. Fitness assays in soil were initiated with 200 µl samples of 1:40 dilutions of the mixtures, mixed briefly by vortexing, and incubated for 96 h at 28°C, 80% relative humidity (Gomez and Buckling, 2011). To recover bacteria from soil 20 glass beads (5 mm diameter) and 10 ml M9 salts (Sambrook et al., 1989) were added to each microcosm and mixed well by vortexing vigorously for 1 min to produce a supernatant that was then sampled for viable numbers as above. Preliminary experiments showed that although this method probably underestimated the total number of bacteria present by approximately 50% (51.6 ± 3%), there was no significant bias in the retrieval of plasmid-free bacteria when compared to bacteria carrying pQBR55, pQBR57 or pQBR103 (see Supporting Information Table S1).

Measurement of conjugation rate (γ) was according to Simonsen et al. (1990): γ = *Ψ* ln(1 + (T / R)(N / D))(1 / (N – N_0_), where *Ψ* is the population growth rate (h^-1^), N_0_ the starting cell density (ml^-1^) and T, R, D and N the end point densities (ml^-1^) of transconjugants, recipients, donors and the total population, respectively. Conjugations were started with a 1:1 mixture of plasmid-bearing *P. fluorescens* SBW25-Gm^R^ donor and plasmid-free *P. fluorescens* SBW25-Sm^R^*lacZ* recipient and grown under conditions similar to the competitive fitness assays. KB agar containing appropriate antibiotics and Hg(II) (20 µM) were used to select plasmid-bearing recipients.

**Plasmid sequencing and analysis**

TruSeq libraries were prepared from 1 µg of DNA per sample with approximately 500 bp insert sizes and sequenced on an Illumina MiSeq with 2x 150 bp paired reads to generate 750–1000 Mbp per sample (ENA project number PRJEB8054). Sequences were assembled using SPAdes v2.5.1 with kmers 21, 33, 55 and 77 (Bankevich et al., 2012) and closed by identifying overlaps between contigs and by PCR (see Supporting Information Document and Figure S2 for details). Putative CDS were identified and annotated using RAST, InterProScan–5, and BLASTP 2.2.28+ (Aziz et al., 2008; Zdobnov and Apweiler, 2001; Altschul et al., 1997). Annotated sequences can be found at the European Nucleotide Archive with the following accession numbers: pQBR44, CDLQ010000001-CDLQ010000002; pQBR55, LN713927; and pQBR57, LN713926. Single nucleotide variants (SNV) and insertions/deletions (indels) were identified using NUCmer/dnadiff (Kurtz et al., 2004). Statistics of GC skew was calculated as (C–G)/(C+G).

**Metabolic profiling**

Metabolic profiling was undertaken using Biolog GN2 MicroPlates as previously described (MacLean et al., 2004). Optical density (OD 660 nm) measurements from duplicate plates were made after 48 h incubation at 28°C, and compared with plasmid-free control strains to determine those carbon sources on which respiration occurred. Since previous work has raised issues over the validity of Biolog assays for such analyses (Leiby and Marx, 2014) we verified candidate substrates as sufficient for growth by measuring the rate of growth (∆OD_600_.second^-1^) of logarithmic-phase M9 minimal medium (Sambrook et al., 1989) cultures containing the test substrate as the sole carbon source at a concentration of 1% w/v.

**Statistics**

For the analysis of conjugation rate, we fitted a linear model where plasmid, growth medium and their interactions were fixed effects. One pQBR103 replicate in soil yielded no transconjugants and was therefore excluded from the analysis and presentation. For the analyses of fitness under different concentrations of Hg(II), data from the highest concentration were not included due to high variance. For both KB and soil competitive fitness assays, the relative fitnesses of plasmid-free bacteria over increasing concentrations of Hg(II) were not found to be significantly different from 1 (KB: intercept = 1.03 ± 0.02, p = 0.2; slope = 0.001 ± 0.003, p = 0.7. Soil: intercept = 0.99 ± 0.02, p = 0.8; slope = –0.004, p = 0.7), so comparisons for each of the plasmid-bearing bacteria were performed against 1. We fitted a linear model where plasmid, mercury and their interactions were fixed effects and relative fitness was the response variable. For soil experiments we used a log transformation of Hg(II) concentration. For the analysis of plasmid-plasmid competitive fitness assays, we fitted a model where competition, mercury, marker orientation and their interactions were fixed effects. Qualitatively similar results were obtained from a mixed effects model, with competition and mercury and their interaction fitted as fixed effects, and marker orientation, nested in competition, fitted as a random effect (not shown). In the soil competitive fitness assays, one pQBR55 replicate at 0.06 µg.g^-1^ had a calculated fitness of zero (no plasmid-bearers were detectable), and two pQBR103 replicates at 64 µg.g^-1^ had calculated fitness of infinite (no plasmid-free were detectable), and were therefore excluded from the analysis and presentation. Statistical analyses were performed using R (R Development Core Team, 2008). Plots were drawn using the ggplot package (H. Wickham, Springer New York, 2009).

**Results**

**Genetic features of the four plasmids**

The salient genetic features of pQBR55, pQBR57 and pQBR44 are shown in Table 1 and Figure 1. Like the previously sequenced and co-occurring pQBR103 (Tett et al., 2007, ENA accession AM235768), pQBR57 was substantially larger than the ~100 kb average size of other sequenced conjugative plasmids (Smillie et al., 2010), while pQBR55 was only slightly larger than average. The mean GC-content of all four pQBR plasmids (52.1–53.8%) was markedly lower than the ~60% found for pseudomonad chromosomes (Winsor et al., 2011).

**Comparative analysis of plasmid sequences**

Consistent with previous findings (Tett et al., 2007), pQBR44 was found to be highly similar to a ~125 kbp fragment of pQBR103 (303,500–4,500 bp, Figure 2). However, pQBR44 did not appear to be a simple deletion variant of pQBR103, for several reasons. First, the Tn5042 mercury resistance (Hg^R^) transposons were located in different positions in the two plasmids. In pQBR103 it was located ~30 kbp downstream of the putative minimal RepA replicon, in pQBR44 it was located ~3 kbp upstream of the homologous RepA region. Second, pQBR44 appears to have acquired a copy of Tn4652, most likely from *P. putida* UWC1 during the capture of plasmids from the rhizoplane (McClure et al., 1989; Lilley et al., 1996; Weinel et al., 2002), and possibly another *P. putida* transposon encoding copper resistance (Supporting Information and Figure S2). Third, despite high similarity between pQBR44 and pQBR103 sequences, a total of 783 single nucleotide variants (SNV) and 48 indels were identified. The greatest divergence was found between pQBR103_0387 and pQBR44_0073, hypothetical proteins with predicted phosphoadenosine phosphosulfate reductase domains which shared only 67% amino acid identity.

Despite having previously been categorised into different RFLP groups (Lilley and Bailey, 1997b), pQBR103 and pQBR57 were found to share distant but extensive similarity across a region of approximately 200 kb (pQBR57 ~10,000–205,000 bp and pQBR103 ~365,000–220,000 bp, see Figure 2 and Supporting Information Figure S3). In this region, similarity was greatest across clusters of genes with predicted functional activity, including a putative chemotaxis phosphorelay system, a type IV pilus, a number of predicted arlysulfatase/S-Adenosyl methionine (‘radical SAM’) enzymes, and genes encoding plasmid conjugative transfer machinery (Tett et al., 2007). However, synteny was incomplete as Tn5042 occupied different positions in the two sequences, and there was an internal region of reduced similarity (pQBR57 120,000–160,000 bp and pQBR103 75,000–180,000 bp). Analysis of the pQBR55 sequence showed that it diverged considerably from pQBR44, pQBR57 and pQBR103, with no extensive backbone similarity to the other pQBR plasmids analysed. However, like pQBR44, pQBR55 had also acquired a copy of Tn4652.

The sequence of the Tn5042 Hg^R^ transposon was identical between the four plasmids, except for a 1 bp non-synonymous substitution in *merR* in pQBR103 and pQBR44. However, Tn5042 was located in different relative positions in the different plasmids, suggesting that it had been horizontally transferred between plasmids at the Wytham site. This suggestion is supported by the fact that each of the Tn5042 sequences have markedly higher GC contents than average for each plasmid (Figure 1), indicating that they have not been subjected to the same biases in DNA replication as the rest of the plasmid sequences (Arakawa and Tomita, 2007).

The pQBR plasmids were similar to other mobile genetic elements isolated from pseudomonads in geographically distant locations (Figure 2 and Supporting Information Figure S3). For example, pQBR57 and pQBR103 had distant similarity to the *P. aeruginosa* pOZ176 antibiotic resistance megaplasmid isolated from a hospital in Guangzhou, China in 2000, (ENA accession KC543497; Xiong et al., 2013). However, pOZ176 uses different *tra*, *par*, and *rep* regions that are found in a region of relatively high GC content with no similarity to the pQBR plasmids. Plasmids pQBR57 and pQBR103 were also found to have high similarity to genomic islands found in *P. stutzeri* B1SMN1 isolated from a lagoon in Menorca in 1988 (ENA accession AMVM00000000; Busquets et al., 2013) and *P. cannabina pv. alisalensis* (*P. syringae pv. maculicola* ES4326), isolated from a diseased radish in Wisconsin, USA in 1965 (ENA accession AEAK00000000; Baltrus et al., 2011). Finally, parts of pQBR55 were similar to the backbone of the catabolic plasmid pCAR1 isolated in *P. resinovorans* from a sewage disposal plant in Japan in 1992 (ENA accession AB088420; Maeda et al., 2003).

**Putative functional regions in the pQBR plasmids**

Replication: Plasmid pQBR44 contains a region 98.5% similar at the nucleotide sequence level to the annotated pQBR103 *rep* region. In contrast, no region similar to the pQBR103 *repA* gene or its origin of replication (*oriV*) could be identified in pQBR57. However, a putative coding sequence (pQBR57_0001) with ~60% amino acid identity to previously annotated *Pseudomonas* TrfA/RepA proteins found 2.5 kbp downstream of a region containing a *dnaA* box (TTATNCACA), 11 copies of a ~80 bp tandem repeat (305,301–306,433 bp), 5 copies of a 132 bp tandem repeat (306,799–131 bp), and an extreme of DNA asymmetry (GC-skew, see Figure 1), may represent the pQBR57 *rep* locus (Mackiewicz, 2004). The pQBR55 *rep* region (ENA accession AJ421512; Turner et al., 2002), identified in other *Pseudomonas* and *Azotobacter* species was clearly identifiable in pQBR55, but not in pQBR44, pQBR57 or pQBR103.

Horizontal transmission: Plasmid pQBR44 appeared to lack the pQBR103 *tra* locus (Figure 2 and Tett et al., 2007) and did not possess any other candidate genes for conjugative transfer. However, it is likely that pQBR44 has lost mobility since its original acquisition, as it was exogenously isolated by conjugation and demonstrated to be transfer-proficient in earlier work (Lilley et al., 1996). Therefore, pQBR44 may represent an example of plasmid lab adaptation occurring outside of a formal experimental evolution study. The pQBR103 *tra* locus (Tett et al., 2007) was part of the region of similarity shared with pQBR57, although the region as a whole (pQBR57 166,906–181,901 bp and pQBR103 170,292–197,117 bp) was only 55.4% similar at the nucleotide level. Candidates for the pQBR57 type-4 coupling protein (T4CP) and major ATPase were identified by comparison with genes from other mobile genetic elements (pQBR57_0202 and pQBR57_0212; Smillie et al., 2010). Two regions of pQBR55 have similarity with the pCAR1 *tra* region, and putative T4CP and major ATPases were identified (pQBR55_0185 and 0173 respectively). For both pQBR57 and pQBR55 the genes best matching these candidates, by BLASTN analysis, were from *Pseudomonas* species.

Vertical transmission: The pQBR plasmids are probably maintained at low copy number, and are likely to encode mechanisms to promote their inheritance such as efficient partition of plasmids between daughter cells (*par* genes) or post-segregational killing of plasmid-free daughters (*psk* or toxin-antitoxin TA genes). A *parAB* locus was identified in pQBR44 that includes the *parA* and *parB* genes as well as the AT-rich *parS* repeat region, which was 99.2% similar to the *par* region of pQBR103 at the nucleotide level (Tett et al., 2007). In contrast, putative *par* regions in pQBR55 and pQBR57 containing predicted *parA* and *parB* genes (pQBR55 78,217–80,003 bp, pQBR55_0121 and 0122; pQBR57 43,015–45,381 bp, pQBR57_0054 and 0055) were quite divergent from pQBR103 *parAB*, and associated *parS* sequences were not found. As with pQBR103, no *psk* or TA systems were identified on pQBR44 or pQBR57. However, pQBR55_0202 and 0203 constitute a likely ChpBS TA system due to their high amino acid identity with ChpBS systems previously identified in a number of other species, including pseudomonads (e.g. Feil et al., 2005). Finally, the divergence between the *par* regions and between the *rep* regions (see previous section) suggests that plasmids pQBR57, pQBR103 and pQBR55 are compatible (Novick, 1987), but a current lack of selectable markers prevents this question from being resolved experimentally.

Accessory functions: All four pQBR plasmids were found to have Tn5042-associated mercury resistance operons which could explain the Hg^R^ phenotype characteristic of this collection of plasmids. Plasmids pQBR55, pQBR57 and pQBR103 also carried putative UV resistance genes (pQBR55_0070 and 0071, pQBR57_0181 and 0182, and pQBR103_0157 and pQBR103_0158), though the pQBR55 and pQBR57 systems may not be functional in *P. fluorescens* SBW25 (Zhang et al., 2004). pQBR57 shared several other regions identified in pQBR103. These included an arylsulfatase/SAM region consisting of 16 genes (73,111–93,469 bp, pQBR57_0084–0099) predicted to encode a number of radical SAM enzymes and cognate regulators that may be involved in biosynthetic processes (Sofia et al., 2001), and a putative type IV pilus region consisting of 10 genes (63,401–73,101 bp, pQBR57_0074–0083), and a chemotaxis operon of 6 genes (28,595–38,795 bp, pQBR57_0044–0049), which might affect host motility. pQBR44 and pQBR55 also contained copies of Tn4652 which is closely related to the toluene degrading transposon Tn4651 (Tsuda and Iino, 1987). Although Tn4652 does not contain the *tol* operon or any identifiable resistance determinants, it includes a putative diacylglycerol kinase and a sulfatase. A full list of putative gene functions identified in these regions is provided in Supporting Information Table S2.

**pQBR55 confers ability to grow on sucrose**

To understand how plasmid carriage affects the host phenotype, pQBR55, pQBR57 and pQBR103 were conjugated into marked strains of the well characterised soil- and plant-associated bacterium *Pseudomonas fluorescens* SBW25, which had been isolated from the same site at Wytham as the pQBR plasmids (Rainey and Bailey, 1996). Consistent with the genetic analyses, and in contrast with earlier work (Lilley et al., 1996), pQBR44 did not transfer and as a result, phenotypic assays were not performed with this plasmid.

First, we tested whether any of the plasmids altered the metabolic ability of their host. Plasmid-carrying and plasmid-free strains were screened using BIOLOG plates and candidate substrates were verified by growth on minimal media containing just one carbon source. Using this approach, *P. fluorescens* SBW25 carrying pQBR55 was found to have gained the ability to grow on sucrose, a potentially beneficial trait in the phytosphere. However, a re-examination of the pQBR55 sequence did not reveal any putative sucrase genes. Although *P. fluorescens* SBW25 is unable to grow on sucrose normally, it encodes a levansucrase (PFLU2294) and is able to catabolise sucrose when the cytoplasmic membrane is disrupted (Spiers and Rainey, 2005). Therefore, pQBR55 may allow sucrose catabolism by encoding a transporter (possibly pQBR55_0083) or by promoting processes that disrupt membrane function.

**Rates of conjugative transfer and segregational loss**

To understand the dynamics of plasmid acquisition and loss, we measured the conjugation rates of each of pQBR55, pQBR57, and pQBR103 between *P. fluorescens* SBW25 hosts in both shaken KB broth and soil microcosms (Figure 3). While pQBR55 and pQBR103 had comparatively low rates of conjugation (Simonsen et al., 1990; Gordon, 1992; Lilley and Bailey, 2002), pQBR57 showed an exceptionally high conjugation rate (effect of plasmid F_2,14_ = 47.633, p < 0.001) and serial passage of pQBR57 conjugation mixtures in Hg(II)-free KB broth found that 100% of the tested recipient population was Hg^R^ after ten transfers (480 hours). Rates of conjugation were found to be slightly higher for all plasmids in soil compared with KB broth (effect of medium F_1,14_ = 14.217, p = 0.002), consistent with other findings showing an increased rate of transfer on surfaces (e.g. Fox et al., 2008).

Plasmid stability was studied by the serial passage of populations of plasmid-carriers in Hg(II)-free KB broth and measuring the loss of Hg^R^. Over ten transfers, few plasmid-free clones were detectable in pQBR55 or pQBR57 cultures suggesting either low rates of segregational loss and/or high rates of horizontal transfer. In contrast, mercury-sensitive (Hg^S^) colonies soon appeared in the pQBR103 populations, indicating that pQBR103 was readily lost by segregation. However, the frequency of pQBR103 carriage remained between 50–75%, suggesting that the plasmid was maintained through frequency-dependent selection and/or horizontal transmission (see Supporting Information Figure S4).

**Carriage of pQBR plasmids confers context dependent fitness effects**

We quantified the effects of carriage on fitness for pQBR55, pQBR57 and pQBR103 by direct competition of plasmid-carrying *P. fluorescens* SBW25-Gm^R^ strains against a plasmid-free *P. fluorescens* SBW25 reference strain. These competitive fitness assays measure the fitness of a test strain relative to that of a reference strain, and therefore the extent to which the test strain is favoured or disfavoured in a given environment. To determine the conditions favouring plasmid bearers we performed assays under different concentrations of Hg(II). Initially, at least three independently isolated transconjugants of each plasmid were tested to assess variation between clones (see Supporting Information Figure S1); subsequent tests (presented below) were performed on one representative transconjugant strain for each plasmid. To account for any effects of the antibiotic resistance marker we also performed control assays with plasmid-free *P. fluorescens* SBW25-Gm^R^ as the test strain.

In the absence of Hg(II), all plasmids imposed fitness costs to their hosts, but costs varied between plasmids: pQBR103 was the most costly (fitness of plasmid-bearing strain relative to plasmid-free at 0 µM Hg(II), 0.54 ± 0.04; one-sample t-test t_3_ = –11.0859, p = 0.002), pQBR55 had intermediate cost (0.73 ± 0.03; t_3_ = –9.3409, p = 0.003), and pQBR57 was the least costly (0.86 ± 0.02, t_3_ = –6.6832, p = 0.007; main effect of plasmid F_2,66_ = 30.14, p<0.001). The fitness of plasmid-bearers relative to plasmid-free cells increased with Hg(II) concentration (main effect of mercury F_1,66_ = 230.90, p<0.001). At high levels of Hg(II) (>30 µM), plasmid-bearers outgrew plasmid-free bacteria, and in some cases, plasmid carriage was essential for growth. The slope of the reaction norm of plasmid-mediated fitness against Hg(II) concentration varied between the different plasmids, with pQBR55 having a significantly shallower slope than either pQBR57 or pQBR103 (effect of mercury × plasmid interaction F_2,66_ = 10.36, p<0.001). At Hg(II) concentrations higher than 20 µM, plasmid-free bacteria were unable to survive at detectable levels in the control assays (i.e. no plasmids in the populations). However, plasmid-free bacteria (i.e. the reference strain) were able to survive in high Hg(II) where plasmid-containing bacteria were present, suggesting that plasmid-free cells were able to exploit the environmental decontamination by plasmid bearers (Ellis et al., 2007). The relationship between the bacterial host and its plasmid symbiont is therefore context-dependent, with the plasmids acting as parasites at low Hg(II) concentrations, and mutualists at high Hg(II) concentrations.

Physically structured soil environments are expected to significantly differ from unstructured experimental liquid microcosms in several ways. For example local concentrations of toxins might be higher in soil, the physiological burden of plasmid carriage might be relatively greater in low-nutrient soil, and the structuring of microbial communities might affect the overall toxicity of Hg(II) (Schlüter, 1993; Platt et al., 2012; Harrison et al., 2007). Therefore, we undertook additional competitive fitness assays in soil microcosms (after Gomez and Buckling, 2011), which provides a physical structure and chemistry more similar to that of natural soil than liquid growth media.

In soil microcosms we observed similar context-dependent fitness effects of plasmid carriage where the fitness of plasmid-bearers relative to plasmid-free strains increased with Hg(II) concentration (effect of mercury F_1,65_ = 68.075, p < 0.001). In contrast to the patterns observed in KB microcosms, pQBR55 was substantially more costly in the soil microcosms in the absence of Hg(II) and at low concentrations (Welch two-sample t-test t_5.66_= –7.6928, p < 0.001). Moreover, concentrations of Hg(II) high enough to select for Hg^R^ plasmids (> 4 µg/g, W ≥ 1) in soil microcosms were non-lethal to the plasmid-free control assays, although the final densities attained in control assays declined with increasing Hg(II) concentration (data not shown). This presumably reflects the non-uniform (patchy) distribution of Hg(II) through the microcosm, resulting in regions where local concentrations were low enough for plasmid-free bacteria to survive and grow.

To test the relative fitness effects of the pQBR plasmids we next measured competitive fitness of pQBR55, pQBR57 and pQBR103 plasmid-bearing *P. fluorescens* SBW25-Gm^R^ strains in direct competition against plasmid-bearing *P. fluorescens* SBW25-Sm^R^*lacZ* strains in all pairwise combinations (three combinations of different competitors × two combinations of markers). Assays were performed in KB and soil microcosms (Figure 5) in the absence of Hg(II), where the carriage of each plasmid is costly, and at high levels of Hg(II) where the plasmids are beneficial to the host.

In KB microcosms, the outcome of plasmid-plasmid competition assays involving pQBR57 was dependent on the presence of Hg(II) (Figure 5a, plasmid × mercury interaction F_2,36_ = 57.782, p <0.001). In particular, pQBR57 performed worse against pQBR55 and pQBR103 when Hg(II) was present compared with assays in Hg-free media. Environmental context therefore changed the fitness hierarchy of the plasmid-bearing bacteria: A mercury resistance plasmid that is more costly than another in the absence of Hg(II) can become more beneficial in Hg(II)-contaminated environments. In this experiment we also detected a significant effect of the antibiotic markers on the outcome of the assay (main effect of marker F_1,36_ = 55.912, p < 0.001, see Supporting Information Figure S5). This effect was likely due to slight variations in fitness between transconjugants with different marker backgrounds (effect of competition × marker orientation F_2,36_ = 19.582, p < 0.001), so precise values for relative fitness cannot be determined (see Supporting Information Figure S5 for details).

In contrast, Hg(II) had no significant effect on the competitive fitness assay in soil microcosms (Figure 5b, plasmid × mercury interaction F_2,33_ = 1.154, p = 0.33). Plasmids varied in competitive fitness (effect of plasmid F_2,33_ = 38.983, p < 0.001) with pQBR55 carriers performing poorly in the soil microcosms, while bacteria carrying pQBR57 and pQBR103 were not significantly different from one another, regardless of the presence or absence of Hg(II).

**Discussion**

Lilley and colleagues isolated a diverse collection of mobilizable mercury resistance plasmids from the same Wytham site over four years (Lilley et al., 1996; Lilley and Bailey, 1997b). The four plasmids that we analysed here came from three RFLP groups, and the greater resolution provided by the full sequence of each has allowed a better determination of the relationships between pQBR103/pQBR44 (Group I), pQBR55 (Group III) and pQBR57 (Group IV), Sequence conservation between these plasmids and other sequences suggests that the plasmid community at Wytham did not diversify independently *in situ*, and is consistent with the hypothesis that the plasmid diversity was ancestral. Locally diverse plasmid assemblages are likely to come about instead by transport over long distances (i.e. immigration), for example by meteorological, hydrological or human-driven processes (Kellogg and Griffin, 2006; Taylor et al., 2011; Wilkinson, 2010).

The high levels of sequence identity between the Tn5042 mercury resistance transposons present in each of the four pQBR plasmids, and the apparent recombination events linking pQBR44 with pQBR103, and pQBR57 and pQBR103 with pOZ176, suggests that recombination may be central to the evolution of mobile genetic elements at different geographical sites. Long distance migration of mobile genetic elements might work in tandem with local recombination events, such as transposition, to facilitate the spread of genes encoding adaptive traits (Frost et al., 2005). Like previous studies of plasmid communities (Sentchilo et al., 2013), the function of the majority of genes identified in the pQBR plasmids is unknown. The fact that many genes from pQBR57 and pQBR103 have also been identified in distantly isolated bacteria (Xiong et al., 2013; Busquets et al., 2013), suggests that they are functional and confer at least occasional fitness advantages to their host bacteria.

The fitness effects of pQBR55, pQBR57 and pQBR103 carriage on the *P. fluorescens* SBW25 host in KB and soil microcosms were found to be dependent on environmental context. As previously shown for pQBR103 (Slater et al., 2008), these plasmids were costly to their bacterial hosts when low levels of Hg(II) were present. However, changes to the environment altered the fitness of plasmid carriers relative to plasmid-free strains: the fitness of plasmid-carrying bacteria increased with Hg(II) levels. Although we performed these experiments with transfer-proficient plasmids, the frequency of transconjugants arising during the assay was relatively low compared to the numbers of competitors, and thus had a negligible effect on the fitness calculations. Similar fitness effects were found when *Escherichia coli* carrying the multi-drug-resistance plasmid pUUH239.2 were challenged with varying concentrations of antibiotics or toxic metals (Gullberg et al., 2014). Fitness has long been known to be a function of genotype and environment, and environmental context has been found to affect both the magnitude and the sign of symbiotic interactions (Wolinska and King, 2009; Chamberlain et al., 2014). Applying this knowledge to plasmid biology defines the symbiotic relationship between plasmids and hosts as a continuum ranging from parasitic to mutualistic depending on environmental challenges and opportunities, with consequences for the ecological persistence and spread of plasmids and the trajectory of coevolution for both parties (Harrison and Brockhurst, 2012).

Mercury detoxification is an active process, causing a local decrease in the concentration of bioactive mercury and making the environment increasingly viable for plasmid-free bacteria (Barkay et al., 2010). At intermediate Hg(II) levels, we found that plasmid-free bacteria often had a fitness advantage over plasmid-bearing bacteria in competitive growth assays, despite the fact that plasmid-free bacteria were unable to survive on their own. Where initial Hg(II) concentration is low enough and initial plasmid frequency is high enough, environmental detoxification probably proceeds sufficiently quickly for plasmid-free bacteria to eventually outcompete plasmid-bearers over the course of the competition. Selection for the plasmid is therefore presumably frequency-dependent under such conditions (Ellis et al., 2007), and more complex fitness measurements may be required to capture the precise dynamics of such a relationship (for example, see Ribeck and Lenski, 2015). It is also interesting to note that unstructured microcosms containing liquid growth medium have shown a greater role here for context-dependency than the more structured soil microcosms. pQBR103-bearing strains are known to have an advantage in Hg(II) contaminated environments, but only when in the close vicinity of Hg(II) sources, with the switch from parasitism to mutualism occurring over just a few hundred micrometers (Slater et al., 2008). Such fine-scale spatial effects may have been missed here in our soil microcosm experiments because fitness was calculated by averaging across the whole microcosm population. In natural soils, Hg(II) distribution is likely to be highly heterogeneous, with retention of Hg(II) affected by numerous physical, chemical and geological factors including pH, mineral composition and temperature (Schlüter, 1993). Such heterogeneity could offer varied microenvironments in which diverse bacteria, in concert with their mobile elements, can coexist.

The effect of Hg(II) concentration on the fitness of plasmid-bearing strains varied between pQBR55, pQBR57 and pQBR103 despite the fact that the mercury resistance operons were almost identical between plasmids. Such differences suggest that there is an undefined interaction between the *mer* operon and other plasmid genes. For example, plasmid-encoded membrane proteins such as conjugative apparatus or the putative pQBR55-encoded sucrose transporter might interfere with Hg(II) import, making detoxification less efficient. Variation in Hg(II) response may help to explain plasmid coexistence at the Wytham site, where environmental heterogeneity could favour different plasmids in different microenvironments. As many antibiotic resistance determinants are also located on and spread via transposons (Partridge et al., 2009), it would be interesting to know the extent to which they share this epistasis.

The bulk concentration of mercury at the Wytham site at the time of plasmid isolation was just 0.05 µg.g^-1^, consistent with a lack of specific contamination and at the lower end of the range for UK rural soils (Morgan et al., 2009). Concentrations of environmental mercury higher than 1 µg.g^-1^ are rare beyond sites of heavy industry or geological disruption (Morgan et al., 2009; Barkay et al., 2010). Our experiments found that far higher concentrations of Hg(II) were required for the pQBR plasmids to confer net fitness benefits to the *P. fluorescens* SBW25 host. Consequently, two outstanding questions remain.

First, if Hg^R^ is not a beneficial trait at the Wytham site, why are there so many mercury resistance plasmids? The relationship between plasmids and Hg^R^ transposons may be quite dynamic, with transposons and plasmids being only transiently associated *in situ*. The different locations of Tn5042 in pQBR44 and pQBR103 suggests that the transposon was acquired independently by the two plasmids, rather than pQBR44 being a deletion-derivative of a pQBR103 Hg^R^ ancestor. Indeed, the accessory gene repertoire of a plasmid may not be constant (Sen et al., 2011; Szczepanowski et al., 2011). Both pQBR44 and pQBR55 appear to have acquired mobile elements from the *P. putida* host used to isolate these plasmids during a relatively short period, and transposition can vary accessory genes between otherwise essentially identical plasmids (e.g. Eikmeyer et al., 2012; Norberg et al., 2014). Like other accessory genes, the genetic basis of mercury resistance is nested in a hierarchy of mobility: the *mer* operon is often associated with transposable elements, which in turn are often on mobilisable and conjugative elements such as plasmids (Partridge et al., 2009; Norman et al., 2009). The pQBR plasmid collection might represent a cross-sectional snapshot of those mobilisable elements that were associated with a Hg^R^ transposon at the Wytham site, and it is interesting to speculate what differences, if any, selection for a different transposon-borne trait might have revealed. The Tn5042 transposon is widespread and highly conserved amongst environmental isolates (Mindlin et al., 2005), and perhaps the diversity of pQBR plasmids speaks to the ability of this transposon to ‘infect’ a broad range of mobilisable and conjugative plasmid backbones, with the plasmids themselves merely ‘hitch-hiking’ when the transposon-borne genes are selected.

Second, if these plasmids are costly to their hosts under Hg(II) conditions approximating those of their natural habitat, how are they able to persist? As suggested above, bulk Hg(II) concentration may be unrepresentative of the local microenvironment experienced by bacteria. However, even if environmental context selected Hg^R^, we have observed transposition of pQBR-based Tn5042 transposons to the host chromosome (unpublished data), suggesting that Hg^R^ plasmids can become redundant (Bergstrom et al., 2000). Furthermore, the decline of pQBR103 in serial cultures demonstrates that plasmids can be lost by segregation. Perhaps the high rate of conjugation of pQBR57 allows it to survive as an infectious agent (Sørensen et al., 2005), although the question of why it carries such a large number of accessory genes remains, since parasites tend to have streamlined genomes (e.g. Tamas et al., 2001).

The putative toxin-antitoxin system identified in pQBR55 may reduce its rate of loss by segregation, although antitoxin genes can also be acquired by bacterial chromosomes, rendering the original plasmid dispensable (Saavedra De Bast et al., 2008). Other plasmid genes might provide benefits to the host in other contexts, such as in close association with plants, or under environmental conditions that fluctuate temporally or are spatially heterogeneous. For example, the pQBR55-conferred ability to catabolise sucrose might be beneficial only during the growing season (Lilley and Bailey, 1997a), with the plasmid becoming costly and poorly maintained at other times, and pQBR103 carriage significantly alters cell physiology and metabolism when grown on different media (Ude et al., 2007).

Perhaps more importantly, plasmids and their hosts can co-evolve, with the costs of plasmid carriage markedly ameliorated by reduction in conjugation rates, loss of plasmid genes, and down-regulation of transcription (Harrison and Brockhurst, 2012). Notably, the GC content of the pQBR plasmids is lower than the *P. fluorescens* SBW25 chromosome, indicating that the pQBR plasmids may be adapted to different hosts in which they are perhaps less costly. Recently it was shown that a costly, unstable non-transmissible plasmid can be quickly stabilised by compensatory chromosomal mutations and transient positive selection for plasmid-encoded genes (San Millan et al., 2014). Moreover, compensation, rather than reversion, tends to be favoured for bacteria which have acquired costly antibiotic resistance mutations (Andersson and Hughes, 2010). Understanding the physiological and genetic mechanisms by which cost of carriage is ameliorated, and the genomic constraints, environmental contexts and community structures that favour amelioration will help resolve the so-called plasmid paradox (Harrison and Brockhurst, 2012).

**Acknowledgements**

We acknowledge the help provided by the staff at the NERC Biomolecular Analysis Facility, University of Liverpool, UK for their assistance with sample preparation and sequencing, as well as Peter Young and the Evolution Discussion Group at the University of York for helpful comments. This work was supported by NERC Standard Grant NE/H005080 and ERC Consolidator Grant Agreement N°311490–COEVOCON to MAB.

**Main figures and table**

**Figure 1.** Ring images of each of the sequenced pQBR plasmids. From the middle of each image, concentric rings show (1) GC content (G + C)/(G + C + A + T). (2) GC skew (C – G)/(G + C). Bars pointing outwards indicate positions where the value was greater than average for that plasmid, and are coloured according to the degree of deviation, with the maximum and minimum indicating deviation of 0.1 from the mean. Values were calculated over sliding windows of 5 kb. (3, 4) Location of predicted CDS on the reverse (anticlockwise) and forward (clockwise) strands respectively. (5) Regions of interest as referred to in the text and Figure 2. Image was drawn using Circos (Krzywinski et al., 2009).

**Figure 2.** Regions of similarity between the pQBR plasmids and with previously sequenced genomes. Linear representations of the pQBR plasmids (right) and four previously sequenced plasmids/genomic islands (right) are shown around the edge of a circle, scaled according to their size (scale in kbp). Note that pQBR44, *P. maculicola* ES4326 and *P. stutzeri* B1SMN1 sequences each consist of multiple contigs as they have not been completely assembled. Semi-transparent connections between the sequences indicate TBLASTX hits filtered by E-value < 1e–1000, pairwise identity > 50% and alignment length > 50 bp. Regions of the sequences discussed in the text are coloured and labelled accordingly. Further information including separate images for each pQBR plasmid is provided in Supporting Information (Table S2, Figure S3). Image was drawn using Circos (Krzywinski et al., 2009).

**Figure 3.** Conjugation rates between *P. fluorescens* SBW25 for each of the three plasmids in KB broth and soil microcosms, calculated according to (Simonsen et al., 1990). Points show means of three (KB) or four (soil) replicates ± SEM.

**Figure 4.** Relative fitness of plasmid-bearing strains when grown in competition with plasmid-free strains in KB broth (left) and potting soil (right) microcosms. Points show means of four replicates ± SEM and are slightly jittered on the x-axis to prevent overplotting. Arrowheads indicate mean fitness > 2.

**Figure 5.** Relative fitness of test plasmid-bearing strains when grown in competition with reference plasmid-bearing strains in KB broth (left) and potting soil (right) microcosms. Points show least square means for assays (n = 8) averaged over experiments and antibiotic markers ± SEM and are slightly jittered on the x-axis to prevent overplotting. Relative fitness for each combination of competitors is plotted for the first named competitor relative to the second.

**Table 1.** Properties of the four sequenced pQBR plasmids. Note that pQBR44 is not fully assembled.

**REFERENCES**

Altschul, S.F., Madden, T.L., Schäffer, A.A., Zhang, J., Zhang, Z., Miller, W., and Lipman, D.J. (1997) Gapped BLAST and PSI-BLAST: a new generation of protein database search programs. *Nucleic Acids Research* **25**: 3389–3402.

Andersson, D.I. and Hughes, D. (2010) Antibiotic resistance and its cost: is it possible to reverse resistance? *Nat Rev Micro* **8**: 260–271.

Arakawa, K. and Tomita, M. (2007) The GC skew index: a measure of genomic compositional asymmetry and the degree of replicational selection. *Evol. Bioinform. Online* **3**: 159–168.

Aziz, R.K., Bartels, D., Best, A.A., DeJongh, M., Disz, T., Edwards, R.A., et al. (2008) The RAST Server: Rapid Annotations using Subsystems Technology. *BMC Genomics* **9**: 75.

Baltrus, D.A. (2013) Exploring the costs of horizontal gene transfer. *Trends in Ecology & Evolution* 28, 489–495.

Baltrus, D.A., Nishimura, M.T., Romanchuk, A., Chang, J.H., Mukhtar, M.S., Cherkis, K., et al. (2011) Dynamic Evolution of Pathogenicity Revealed by Sequencing and Comparative Genomics of 19 Pseudomonas syringae Isolates. *PLoS Pathog* **7**: e1002132.

Bankevich, A., Nurk, S., Antipov, D., Gurevich, A.A., Dvorkin, M., Kulikov, A.S., et al. (2012) SPAdes: A New Genome Assembly Algorithm and Its Applications to Single-Cell Sequencing. *Journal of Computational Biology* **19**: 455–477.

Barkay, T., Kritee, K., Boyd, E., and Geesey, G. (2010) A thermophilic bacterial origin and subsequent constraints by redox, light and salinity on the evolution of the microbial mercuric reductase. *Environmental Microbiology* **12**: 2904–2917.

Bergstrom, C.T., Lipsitch, M., and Levin, B.R. (2000) Natural selection, infectious transfer and the existence conditions for bacterial plasmids. *Genetics* **155**: 1505–1519.

Brown Kav, A., Sasson, G., Jami, E., Doron-Faigenboim, A., Benhar, I., and Mizrahi, I. (2012) Insights into the bovine rumen plasmidome. *Proceedings of the National Academy of Sciences* **109**: 5452–5457.

Brown, C.J., Sen, D., Yano, H., Bauer, M.L., Rogers, L.M., Van der Auwera, G.A., and Top, E.M. (2013) Diverse Broad-Host-Range Plasmids from Freshwater Carry Few Accessory Genes. *Applied and Environmental Microbiology* **79**: 7684–7695.

Busquets, A., Pena, A., Gomila, M., Mayol, J., Bosch, R., Nogales, B., et al. (2013) Draft Genome Sequence of Pseudomonas stutzeri Strain B1SMN1, a Nitrogen-Fixing and Naphthalene-Degrading Strain Isolated from Wastewater. *Genome Announcements* **1**: e00584–13–e00584–13.

Chamberlain, S.A., Bronstein, J.L., and Rudgers, J.A. (2014) How context dependent are species interactions? *Ecology Letters* **17**: 881–890.

Dahlberg, C. and Chao, L. (2003) Amelioration of the cost of conjugative plasmid carriage in Eschericha coli K12. *Genetics* **165**: 1641–1649.

De Gelder, L., Ponciano, J.M., Joyce, P., and Top, E.M. (2007) Stability of a promiscuous plasmid in different hosts: no guarantee for a long-term relationship. *Microbiology* **153**: 452–463.

Eikmeyer, F., Hadiati, A., Szczepanowski, R., Wibberg, D., Schneiker-Bekel, S., Rogers, L.M., et al. (2012) The complete genome sequences of four new IncN plasmids from wastewater treatment plant effluent provide new insights into IncN plasmid diversity and evolution. *Plasmid* **68**: 13–24.

Ellis, R.J., Lilley, A.K., Lacey, S.J., Murrell, D., and Godfray, H.C.J. (2007) Frequency-dependent advantages of plasmid carriage by Pseudomonas in homogeneous and spatially structured environments. *The ISME Journal* **1**: 92–95.

Feil, H., Feil, W.S., Chain, P., Larimer, F., DiBartolo, G., Copeland, A., et al. (2005) Comparison of the complete genome sequences of Pseudomonas syringae pv. syringae B728a and pv. tomato DC3000. *Proc. Natl. Acad. Sci. U.S.A.* **102**: 11064–11069.

Fox, R.E., Zhong, X., Krone, S.M., and Top, E.M. (2008) Spatial structure and nutrients promote invasion of IncP-1 plasmids in bacterial populations. *The ISME Journal* **2**: 1024–1039.

Frost, L.S., Leplae, R., Summers, A.O., and Toussaint, A. (2005) Mobile genetic elements: the agents of open source evolution. *Nat Rev Micro* **3**: 722–732.

Gogarten, J. P. & Townsend, J. P. (2005) Horizontal gene transfer, genome innovation and evolution. *Nat Rev Micro* **3,** 679–687.

Gomez, P. and Buckling, A. (2011) Bacteria-Phage Antagonistic Coevolution in Soil. *Science* **332**: 106–109.

Gordon, D.M. (1992) Rate of plasmid transfer among Escherichia coli strains isolated from natural populations. *Microbiology* **138**: 17–21.

Gullberg, E., Albrecht, L.M., Karlsson, C., Sandegren, L., and Andersson, D.I. (2014) Selection of a Multidrug Resistance Plasmid by Sublethal Levels of Antibiotics and Heavy Metals. *mBio* **5**: e01918–14–e01918–14.

Harrison, E. and Brockhurst, M.A. (2012) Plasmid-mediated horizontal gene transfer is a coevolutionary process. *Trends in Microbiology* **20**: 262–267.

Harrison, J.J., Ceri, H., and Turner, R.J. (2007) Multimetal resistance and tolerance in microbial biofilms. *Nat Rev Micro* **5**: 928–938.

Heuer, H. & Smalla, K. (2012) Plasmids foster diversification and adaptation of bacterial populations in soil. *FEMS Microbiol Rev* **36**: 1083–1104.

Kellogg, C.A. and Griffin, D.W. (2006) Aerobiology and the global transport of desert dust. *Trends in Ecology & Evolution* **21**: 638–644.

Krzywinski, M., Schein, J., Birol, I., Connors, J., Gascoyne, R., Horsman, D., et al. (2009) Circos: an information aesthetic for comparative genomics. *Genome Res.* **19**: 1639–1645.

Kurtz, S., Phillippy, A., Delcher, A.L., Smoot, M., Shumway, M., Antonescu, C., and Salzberg, S.L. (2004) Versatile and open software for comparing large genomes. *Genome Biol* **5**: R12.

Lambertsen, L., Sternberg, C., and Molin, S. (2004) Mini-Tn7 transposons for site-specific tagging of bacteria with fluorescent proteins. *Environmental Microbiology* **6**: 726–732.

Leiby, N. & Marx, C. J. Metabolic Erosion Primarily Through Mutation Accumulation, and Not Tradeoffs, Drives Limited Evolution of Substrate Specificity in *Escherichia coli*. *Plos Biol* **12,** e1001789 (2014).

Lenski, R.E., Rose, M.R., Simpson, S.C., and Tadler, S.C. (1991) Long-term experimental evolution in Escherichia coli. I. Adaptation and divergence during 2,000 generations. *American Naturalist* 1315–1341.

Lilley, A.K. and Bailey, M.J. (1997a) Impact of Plasmid pQBR103 Acquisition and Carriage on the Phytosphere Fitness of Pseudomonas fluorescens SBW25: Burden and Benefit. *Applied and Environmental Microbiology* **63**: 1584–1587.

Lilley, A.K. and Bailey, M.J. (1997b) The acquisition of indigenous plasmids by a genetically marked pseudomonad population colonizing the sugar beet phytosphere is related to local environmental conditions. *Applied and Environmental Microbiology* **63**: 1577–1583.

Lilley, A.K. and Bailey, M.J. (2002) The transfer dynamics of Pseudomonas sp. plasmid pQBR11 in biofilms. *FEMS Microbiology Ecology* **42**: 243–250.

Lilley, A.K., Bailey, M.J., Day, M.J., and Fry, J.C. (1996) Diversity of mercury resistance plasmids obtained by exogenous isolation from the bacteria of sugar beet in three successive years. *FEMS Microbiology Ecology* **20**: 211–227.

Lilley, A.K., Fry, J.C., Day, M.J., and Bailey, M.J. (1994) In situ transfer of an exogenously isolated plasmid between Pseudomonas spp. in sugar beet rhizosphere. *Microbiology* **140**: 27–33.

Mackiewicz, P. (2004) Where does bacterial replication start? Rules for predicting the oriC region. *Nucleic Acids Research* **32**: 3781–3791.

MacLean, R.C., Bell, G., and Rainey, P.B. (2004) The evolution of a pleiotropic fitness tradeoff in Pseudomonas fluorescens. *Proc. Natl. Acad. Sci. U.S.A.* **101**: 8072–8077.

Maeda, K., Nojiri, H., Shintani, M., Yoshida, T., Habe, H., and Omori, T. (2003) Complete Nucleotide Sequence of Carbazole/Dioxin-degrading Plasmid pCAR1 in Pseudomonas resinovorans Strain CA10 Indicates its Mosaicity and the Presence of Large Catabolic Transposon Tn4676. *Journal of Molecular Biology* **326**: 21–33.

McClure, N.C., Weightman, A.J., and Fry, J.C. (1989) Survival of Pseudomonas putida UWC1 containing cloned catabolic genes in a model activated-sludge unit. *Applied and Environmental Microbiology* **55**: 2627–2634.

Mindlin, S., Minakhin, L., Petrova, M., Kholodii, G., Minakhina, S., Gorlenko, Z., and Nikiforov, V. (2005) Present-day mercury resistance transposons are common in bacteria preserved in permafrost grounds since the Upper Pleistocene. *Research in Microbiology* **156**: 994–1004.

Morgan, H., De Búrca, R., Martin, I., and Jeffries, J. (2009) Soil Guideline Values for mercury in soil. *Environment Agency Science Report SC050021*. Environment Agency, Bristol, UK.

Morton, E.R., Platt, T.G., Fuqua, C., and Bever, J.D. (2014) Non-additive costs and interactions alter the competitive dynamics of co-occurring ecologically distinct plasmids. *Proceedings of the Royal Society B: Biological Sciences* **281**: 20132173–20132173.

Novick, R. P. (1987) Plasmid incompatibility. *Microbiol. Rev.* **51,** 381–395.

Norberg, P., Bergström, M. & Hermansson, M. (2014) Complete Nucleotide Sequence and Analysis of Two Conjugative Broad Host Range Plasmids from a Marine Microbial Biofilm. *PLoS ONE* **9**, e92321

Norman, A., Hansen, L.H., and Sorensen, S.J. (2009) Conjugative plasmids: vessels of the communal gene pool. *Philosophical Transactions of the Royal Society B: Biological Sciences* **364**: 2275–2289.

Platt, T.G., Bever, J.D., and Fuqua, C. (2012) A cooperative virulence plasmid imposes a high fitness cost under conditions that induce pathogenesis. *Proceedings of the Royal Society B: Biological Sciences* **279**: 1691–1699.

Rainey, P.B. and Bailey, M.J. (1996) Physical and genetic map of the Pseudomonas fluorescens SBW25 chromosome. *Mol. Microbiol.* **19**: 521biol.

Ribeck, N. & Lenski, R. E. Modeling and quantifying frequency-dependent fitness in microbial populations with cross-feeding interactions. *Evolution* (2015). doi:10.1111/evo.12645

Saavedra De Bast, M., Mine, N., and Van Melderen, L. (2008) Chromosomal Toxin-Antitoxin Systems May Act as Antiaddiction Modules. *Journal of Bacteriology* **190**: 4603–4609.

Sambrook, J., Fritsch, E.F., Maniatis, T., others (1989) Molecular Cloning. Cold Spring Harbor Laboratory Press, New York.

San Millan, A., Peña-Miller, R., Toll-Riera, M., Halbert, Z.V., McLean, A.R., Cooper, B.S., and MacLean, R.C. (2014) Positive selection and compensatory adaptation interact to stabilize non-transmissible plasmids. *Nature Communications* **5**: 5208.

Schlüter, K. (1993) The fate of mercury in soil. A review of current knowledge. Soil and Groundwater Research Report IV *Tech. Rep. EUR 14666 EN*. Commission of the European Communities, Luxembourg, 1993.

Sen, D., Van der Auwera, G.A., Rogers, L.M., Thomas, C.M., Brown, C.J., and Top, E.M. (2011) Broad-Host-Range Plasmids from Agricultural Soils Have IncP-1 Backbones with Diverse Accessory Genes. *Applied and Environmental Microbiology* **77**: 7975–7983.

Sentchilo, V., Mayer, A.P., Guy, L., Miyazaki, R., Tringe, S.G., Barry, K., et al. (2013) Community-wide plasmid gene mobilization and selection. *The ISME Journal* 1–14.

Simonsen, L., Gordon, D.M., Stewart, F.M., and Levin, B.R. (1990) Estimating the rate of plasmid transfer: an end-point method. *Microbiology (Reading, Engl.)* **136**: 2319–2325.

Slater, F.R., Bruce, K.D., Ellis, R.J., Lilley, A.K., and Turner, S.L. (2008) Heterogeneous Selection in a Spatially Structured Environment Affects Fitness Tradeoffs of Plasmid Carriage in Pseudomonads. *Applied and Environmental Microbiology* **74**: 3189–3197.

Smillie, C., Garcillan-Barcia, M.P., Francia, M.V., Rocha, E.P.C., and la Cruz, de, F. (2010) Mobility of Plasmids. *Microbiology and Molecular Biology Reviews* **74**: 434–452.

Smit, E., Wolters, A., and van Elsas, J.D. (1998) Self-transmissible mercury resistance plasmids with gene-mobilizing capacity in soil bacterial populations: influence of wheat roots and mercury addition. *Applied and Environmental Microbiology* **64**: 1210–1219.

Sofia, H.J., Chen, G., Hetzler, B.G., Reyes-Spindola, J.F., and Miller, N.E. (2001) Radical SAM, a novel protein superfamily linking unresolved steps in familiar biosynthetic pathways with radical mechanisms: functional characterization using new analysis and information visualization methods. *Nucleic Acids Research* **29**: 1097–1106.

Spiers, A.J. and Rainey, P.B. (2005) The Pseudomonas fluorescens SBW25 wrinkly spreader biofilm requires attachment factor, cellulose fibre and LPS interactions to maintain strength and integrity. *Microbiology (Reading, Engl.)* **151**: 2829–2839.

Szczepanowski, R., Eikmeyer, F., Harfmann, J., Blom, J., Rogers, L.M., Top, E.M., and Schlüter, A. (2011) Journal of Biotechnology. *Journal of Biotechnology* **155**: 95–103.

Sørensen, S.J., Bailey, M., Hansen, L.H., Kroer, N., and Wuertz, S. (2005) Studying plasmid horizontal transfer in situ: a critical review. *Nat Rev Micro* **3**: 700–710.

Tamas, I., Klasson, L.M., Sandström, J.P., and Andersson, S.G. (2001) Mutualists and parasites: how to paint yourself into a (metabolic) corner. *FEBS Lett.* **498**: 135–139.

Taylor, N.G.H., Verner-Jeffreys, D.W., and Baker-Austin, C. (2011) Aquatic systems: maintaining, mixing and mobilising antimicrobial resistance? *Trends in Ecology & Evolution* **26**: 278–284.

Tett, A., Spiers, A.J., Crossman, L.C., Ager, D., Ciric, L., Dow, J.M., et al. (2007) Sequence-based analysis of pQBR103; a representative of a unique, transfer-proficient mega plasmid resident in the microbial community of sugar beet. *The ISME Journal* **1**: 331–340.

Toussaint, A. and Merlin, C. (2002) Mobile Elements as a Combination of Functional Modules. *Plasmid* **47**: 26–35.

Tsuda, M. & Iino, T. (1987) Genetic analysis of a transposon carrying toluene degrading genes on a TOL plasmid pWW0. *Molecular and General Genetics* **210,** 270–276.

Turner, S.L., Lilley, A.K., and Bailey, M.J. (2002) Two dnaB genes are associated with the origin of replication of pQBR55, an exogenously isolated plasmid from the rhizosphere of sugar beet. *FEMS Microbiology Ecology* **42**: 209–215.

Ude, S., Bailey, M.J., Huang, W.E., and Spiers, A.J. (2007). The environmental plasmid pQBR103 alters the single-cell Raman spectral profile of *Pseudomonas fluorescens* SBW25. *Microbial Ecology* **53:** 494–497.

Viegas, C.A., Lilley, A.K., Bruce, K., and Bailey, M.J. (1997) Description of a novel plasmid replicative origin from a genetically distinct family of conjugative plasmids associated with phytosphere microflora. *FEMS Microbiology Letters* **149**: 121–127.

Weinel, C., Nelson, K.E., and Tümmler, B. (2002) Global features of the Pseudomonas putida KT2440 genome sequence. *Environmental Microbiology* **4**: 809–818.

Wilkinson, D.M. (2010) Have we underestimated the importance of humans in the biogeography of free-living terrestrial microorganisms? *Journal of Biogeography* **37**: 393–397.

Winsor, G.L., Lam, D.K.W., Fleming, L., Lo, R., Whiteside, M.D., Yu, N.Y., et al. (2011) Pseudomonas Genome Database: improved comparative analysis and population genomics capability for Pseudomonas genomes. *Nucleic Acids Research* **39**: D596–600.

Wolinska, J. and King, K.C. (2009) Environment can alter selection in host–parasite interactions. *Trends in Parasitology* **25**: 236–244.

Xiong, J., Alexander, D.C., Ma, J.H., Deraspe, M., Low, D.E., Jamieson, F.B., and Roy, P.H. (2013) Complete Sequence of pOZ176, a 500-Kilobase IncP-2 Plasmid Encoding IMP-9-Mediated Carbapenem Resistance, from Outbreak Isolate Pseudomonas aeruginosa 96. *Antimicrobial Agents and Chemotherapy* **57**: 3775–3782.

Zdobnov, E.M. and Apweiler, R. (2001) InterProScan—an integration platform for the signature-recognition methods in InterPro. *Bioinformatics* **17**: 847–848.

Zhang, X.-X. and Rainey, P.B. (2007) Construction and validation of a neutrally-marked strain of Pseudomonas fluorescens SBW25. *Journal of Microbiological Methods* **71**: 78–81.

Zhang, X.-X., Lilley, A.K., Bailey, M.J., and Rainey, P.B. (2004) The indigenous Pseudomonas plasmid pQBR103 encodes plant-inducible genes, including three putative helicases. *FEMS Microbiology Ecology* **51**: 9–17.
